# Local Membrane Deformations Activate Ca^2+^-Dependent K^+^ and Anionic Currents in Intact Human Red Blood Cells

**DOI:** 10.1371/journal.pone.0009447

**Published:** 2010-02-26

**Authors:** Agnieszka Dyrda, Urszula Cytlak, Anna Ciuraszkiewicz, Agnieszka Lipinska, Anne Cueff, Guillaume Bouyer, Stéphane Egée, Poul Bennekou, Virgilio L. Lew, Serge L. Y. Thomas

**Affiliations:** 1 Centre National de la Recherche Scientifique – Université Pierre et Marie Curie Paris6, UMR 7150, Roscoff, France; 2 Institute of Physics, University of Technology, Wroclaw, Poland; 3 Institute of Biology, University of Copenhagen, Copenhagen, Denmark; 4 Department of Physiology Development and Neuroscience, University of Cambridge, Cambridge, United Kingdom; INSERM U567, Institut Cochin, France

## Abstract

**Background:**

The mechanical, rheological and shape properties of red blood cells are determined by their cortical cytoskeleton, evolutionarily optimized to provide the dynamic deformability required for flow through capillaries much narrower than the cell's diameter. The shear stress induced by such flow, as well as the local membrane deformations generated in certain pathological conditions, such as sickle cell anemia, have been shown to increase membrane permeability, based largely on experimentation with red cell suspensions. We attempted here the first measurements of membrane currents activated by a local and controlled membrane deformation in single red blood cells under on-cell patch clamp to define the nature of the stretch-activated currents.

**Methodology/Principal Findings:**

The cell-attached configuration of the patch-clamp technique was used to allow recordings of single channel activity in intact red blood cells. Gigaohm seal formation was obtained with and without membrane deformation. Deformation was induced by the application of a negative pressure pulse of 10 mmHg for less than 5 s. Currents were only detected when the membrane was seen domed under negative pressure within the patch-pipette. K^+^ and Cl^−^ currents were strictly dependent on the presence of Ca^2+^. The Ca^2+^-dependent currents were transient, with typical decay half-times of about 5–10 min, suggesting the spontaneous inactivation of a stretch-activated Ca^2+^ permeability (PCa). These results indicate that local membrane deformations can transiently activate a Ca^2+^ permeability pathway leading to increased [Ca^2+^]_i_, secondary activation of Ca^2+^-sensitive K^+^ channels (Gardos channel, IK1, KCa3.1), and hyperpolarization-induced anion currents.

**Conclusions/Significance:**

The stretch-activated transient PCa observed here under local membrane deformation is a likely contributor to the Ca^2+^-mediated effects observed during the normal aging process of red blood cells, and to the increased Ca^2+^ content of red cells in certain hereditary anemias such as thalassemia and sickle cell anemia.

## Introduction

The cell-attached configuration of the patch-clamp technique respects the integrity of the intracellular milieu. It therefore reflects best the physiological condition of the currents recorded across the membrane patch trapped within the tip of the microelectrode. Patch-clamping of human red cells in this configuration is particularly difficult because of the extreme fragility of the cell membrane, which accounts for the scarcity of the literature on its application to study channel activity in intact red cells [1], [2], [3], [4], [5].

Although a negative pressure pulse of about 10 mmHg is usually applied to establish GigaOhm seals at the tip of the patch pipette, comparable good seals can be obtained without underpressure, albeit with a lower success rate. Once the seal is established, the membrane deformation induced by the glass pipette, regardless of the intensity of underpressure, is not under experimental control, and may vary from one cell to another.

In our previous studies in intact red cells we seldom observed spontaneous channel activity in cell attached patches when the cells were bathed in physiological saline solution. Episodically though, we did detect transient activity immediately following seal formation, but only when contact was facilitated by underpressure. Intrigued by the systematic link between the negative pressure pulse and the transient current response, we explored some of the medium requirements in preliminary experiments. It soon became clear that the presence of Ca^2+^ in the bathing medium was essential for the transient current response.

The association between red cell membrane deformation and changes in membrane permeability affecting Ca^2+^ and other ions has been documented for a number of physiological and pathological processes in the past, based mostly on experimentation with red blood cell suspensions. Physiological shear stress in the circulation has been claimed to cause a reversible increase in Ca^2+^ permeability[6], [7], [8], [9], [10]. Recent evidence supported the view that the increasing density of aging human RBCs, attributed to a progressive loss of KCl and osmotic water, results from the cumulative effects of declining Ca^2+^ extrusion capacity of the plasma membrane Ca^2+^ pump, aided by minor episodes of increased Ca^2+^ permeability in the circulation [11], [12], [13], [14], [15]. In sickle cell anemia, deoxygenation of red blood cells in the circulation reversibly increases their membrane permeability to Na^+^, K^+^, Ca^2+^ and Mg^2+^ [16], [17], [18], [19], [20], [21], [22], and this increase has been attributed to the activation of Psickle, a poorly selective cation permeability pathway thought to be generated by the protruding deformation of the RBC membrane on contact with polymers of deoxy-hemoglobin S [22], [23]. The increase in [Ca^2+^]_i_ resulting from Psickle activation in turn activates the Ca^2+^-sensitive K^+^ channel of the red cell membrane (Gardos channel, IK1, KCa3.1)[24], [25] a critical stage in the mechanism of sickle cell dehydration [22]. A localized increase in red cell Ca^2+^ associated with local dynamic membrane deformations was also suggested to be involved in the process of apical alignment of malaria merozoites, just before invasion [13].

There is a suggestive link between our observations on deformation-induced red cell currents and the body of research briefly reviewed above. The potential relevance of this link prompted the current investigation. The electrophysiological evidence presented here shows that seal formation, when associated with negative pressure, causes transient activation of Gardos channels and anion currents, and that this phenomenon results from activation of a permeability pathway with a finite Ca^2+^ conductance (PCa) and spontaneous decay.

## Results

In the experimental series reported here, over 500 attempts were made to obtain a GigaOhm seal in cell attach configuration, among which 284 were successful. The mean duration of the high-resistance cell-attached seals was 25–35 min, allowing multiple sequences of 1 min recording. The noise level remained within the ±0.08 pA range, ensuring optimal signal to noise ratios in the detection and measurement of channel-mediated currents in the red cell membrane. Channel conductances were measured by applying alternating ramps of 1 min duration of hyperpolarizing or depolarizing voltages, but most other current measurements were performed at the physiological spontaneous membrane potential of RBCs (∼−10 mV).

### Evidence for Spontaneous Channel Activity Immediately after Seal Formation

**Figure 1:**
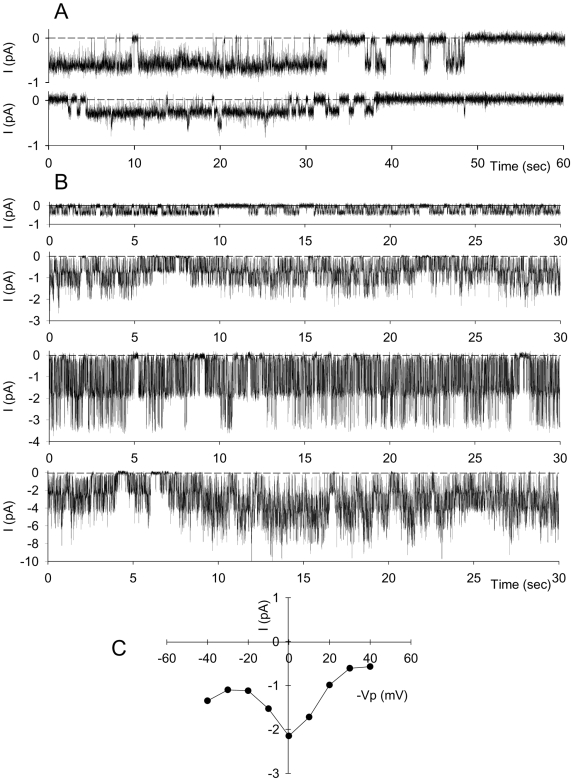
Evidence for spontaneous channel activity following seal formation. Patch-clamp single-channel recordings of red blood cell spontaneous membrane electrical activity (*i.e.* in absence of pipette potential: -Vp = 0 mV) indicating an inwardly directed movement of cations or an outward movement of anions during the first 10 min following seal formation. Seals were obtained by underpressure inducing membrane deformation in the patch pipette. Panel **A** shows two typical recordings obtained with solution A (150 mM NaCl, pCa3) in the pipette and bathing solutions. Long channel openings are followed by long silent intervals. (**B**) shows four different recordings, obtained with solution B (150 mM KCl, pCa3) in the pipette solution and solution A (150 mM NaCl, pCa 3) in bathing solution. Dashed lines indicate the baseline corresponding to the closed state. Panel **C** presents an example of I/V plot obtained when 1 min ramps of depolarizng or hyperpolarizing voltages were imposed in the pipette in the following order: (in mV) 0, −10, +10, −20, +20, −30, +30, −40, +40, immediately following seal formation. This V-shaped I/V curve suggests a continuous decline with time in channel activity.

Using the cell-attached configuration with solution A (150 mM NaCl, pCa = 3) in the pipette and bathing solutions (56 patches), RBC membranes seldom displayed channel activity when recordings were performed more than 10–15 min after seal formation (5 out of 56), at the spontaneous membrane potential, *i.e.* at pipette potential of zero mV (-Vp = 0 mV), whatever the degree of membrane deformation induced by underpressure. However, recordings made during the first ∼10 min following seal formation frequently displayed inward current at the spontaneous membrane potential (41 out of 56), corresponding to long channel openings followed by long silent intervals, as shown with two examples in Figure 1A.

In contrast, when NaCl was replaced by KCl (solution B, pCa = 3) in the pipette solution, channel activity was always observed immediately after seal formation as bursts of channel openings separated by brief closures, and all patches (n = 136) contained one or more active channels (Figure 1B). The size of the current recorded at -Vp = 0 mV varied from one cell to another in the range −0.30 pA to −2.5 pA (see four representative examples in Figure 1B) indicating an inwardly directed movement of cations or an outward movement of anions. Although the presence of spontaneous channel activity correlated closely with the application of negative pressure during, or after seal formation, the mean size of the recorded current was not correlated with the magnitude of the underpressure imposed (data not shown).

### Characterization of the Varied Time-Course and Pattern of the Recorded Currents

A protocol of hyperpolarizing and depolarizing voltage ramps was performed to characterize the conductance properties of the measured currents. Sixty seconds of current recordings were obtained for each imposed voltage, in the following order (in mV): 0, ±10, ±20, …, ±100. With this protocol we systematically obtained unusual V-shaped I/V curves (Figure 1C) which could be explained by assuming a continuous decline with time in channel activity, whatever the value of the potential imposed to the patch membrane.

**Figure 2 pone-0009447-g002:**
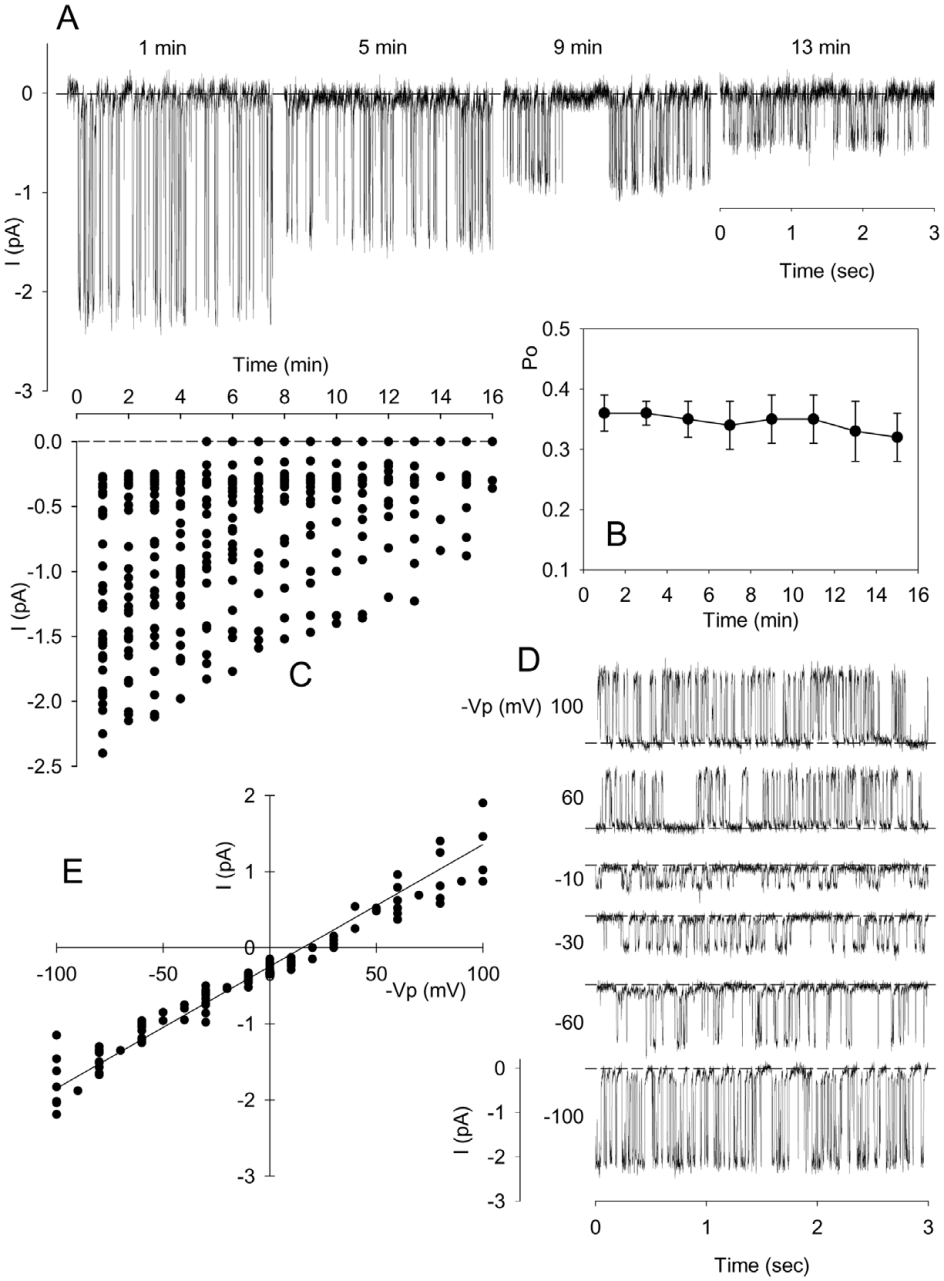
Transient nature of recorded currents. Panel **A** provides an example (representative of 50 patches) of progressive decline of a large channel activity recorded at different times after seal formation with solution B (150 mM KCl, pCa3) in the pipette solution and solution A (150 mM NaCl, pCa3) in bathing solution at 0 mV pipette potential. Panel **B** displays the corresponding mean (n = 8) evolution of the open probability (Po) calculated from recordings displaying only one channel. Panel **C** summarizes the evolution of currents recorded from 35 separate patches during the first 16 min. Once the current was stable in the range 0.3–0.5 pA, the currents obtained by evoking a series of test potentials from −100 to +100 mV for 15 sec from a holding potential of 0 mV were recorded as shown in the example of Panel **D** and the corresponding I/V pairs were collected in the I/V plot displayed in Panel **E** (127 points from 14 experiments).

To confirm this suspected time-decline and to investigate its modality, currents were recorded continuously for up to 16 min in 35 different patches (Figure 2). The results in Figure 2A show a representative sample of the time-course and pattern of decline observed in this experimental series. At least during the first 10 min, open state probability (Po) remained unchanged, within the range 30% to 40% (Figure 2B). These results support the explanation of the unusual V-shaped I/V pattern in terms of a rapid spontaneous decline in channel activity during the course of the I/V measuring protocol. Figure 2C shows minute by minute the evolution with time of the current level recorded at 0 mV in the 35 different patches. During the first five minutes all patches exhibited channel activity in the range 0.3 to 2.5 pA. After this time, an increasing number of patches lost activity whilst the remaining continued a progressive decline which never extended beyond 17 minutes after seal formation.

When the declining currents reached the 0.3–0.5 pA level their magnitude remained quite constant for a few minutes but their Po declined progressively before total loss of channel activity. The duration of this period varied considerably among cells. The currents recorded at different imposed potentials during this pre-extinction period are shown in Figure 2D. The I/V records from 14 experiments, measured in the voltage range −100 mV to +100 mV in alternating ±10 mV steps are shown in Figure 2E. The results show no significant deviation from linearity and the mean ohmic channel conductance from the linear regression fit was 14.5 pS.

**Figure 3:**
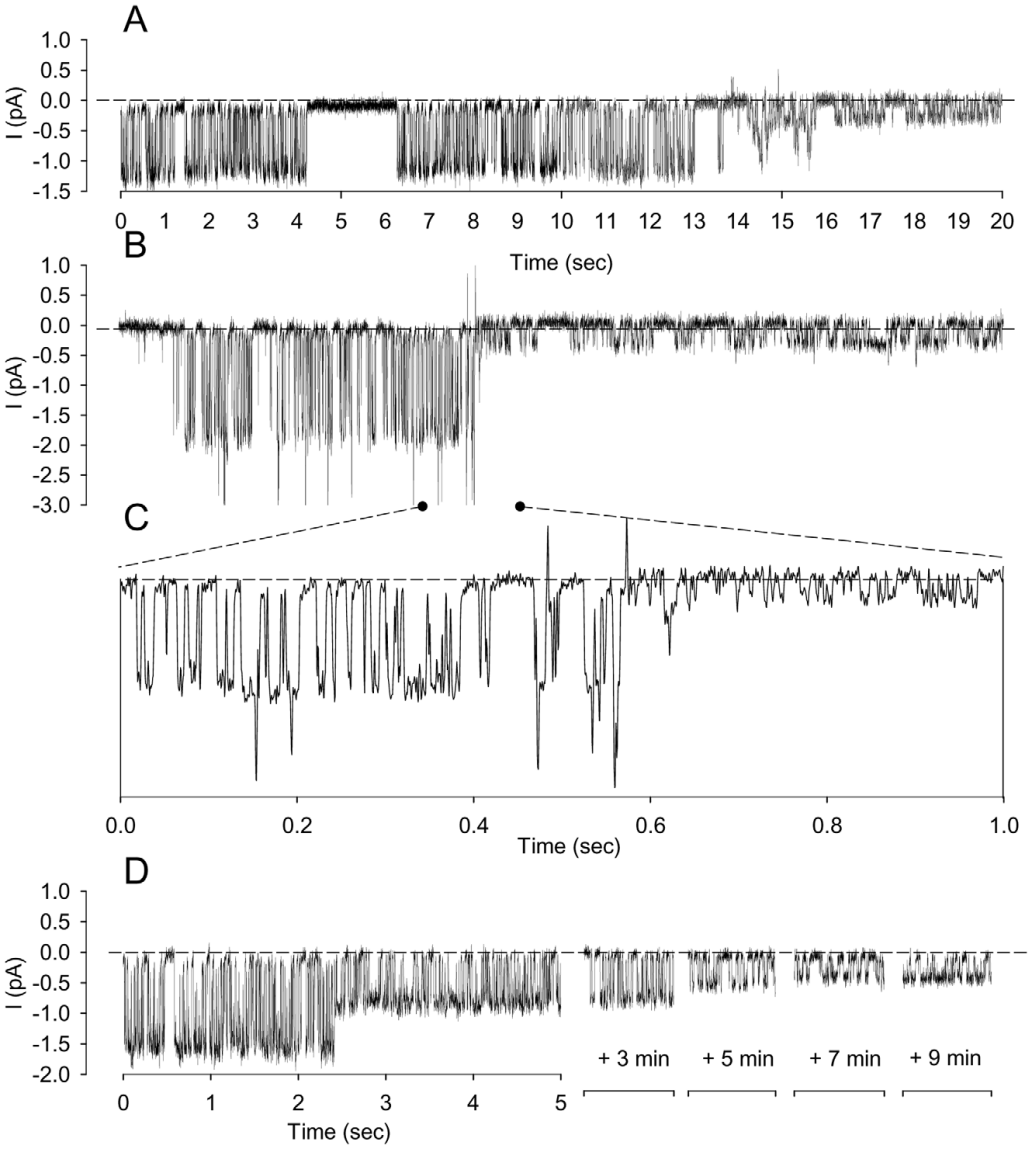
Patterns of channel activity decline. Three fast modalities of current transitions could be discerned during activity decline: Panel **A** (representative of 6 recordings), fast (3 sec) but progressive transition from a large current (1.4 pA in the present example) to a stable 0.3–0.5 pA current; Panel **B** and **C** (representative of 12 recordings), instantaneous transition from large (1.8 pA in the present example) to a stable 0.3–0.5 pA current; Panel **D** (representative of 9 recordings), instantaneous transition from a large (1.7 pA in the present example) to an intermediary current (0.9 pA) which thereafter followed progressive decline to a stable 0.3–0.5 pA current. In all cases recordings were obtained with solution A (150 mM NaCl, pCa3) in the bathing solutions and solution B (150 mM KCl, pCa3) in the patch pipettes.

In 27 out of 136 patches we could record very fast transitions in channel activity instead of progressive decline. Figure 3 illustrates the observed variety of fast transition patterns. These can be grouped within three broad modalities: i) Rapid (3 sec) but gradual transition from a high current to the pre-extinction level (0.3–0.5 pA) (Figure 3A, n = 6), ii) Instant transition to the 0.3–0.5 pA level (Figures 3B and 3C, n = 12), and iii) Instant transitions from high to intermediate and low current levels (Figure 1D, n = 9). In multi-channel patches, the independent expression of these different patterns could generate very complex records with large and small currents superimposed.

### Identification of Gardos Channel-Mediated Currents

**Figure 4:**
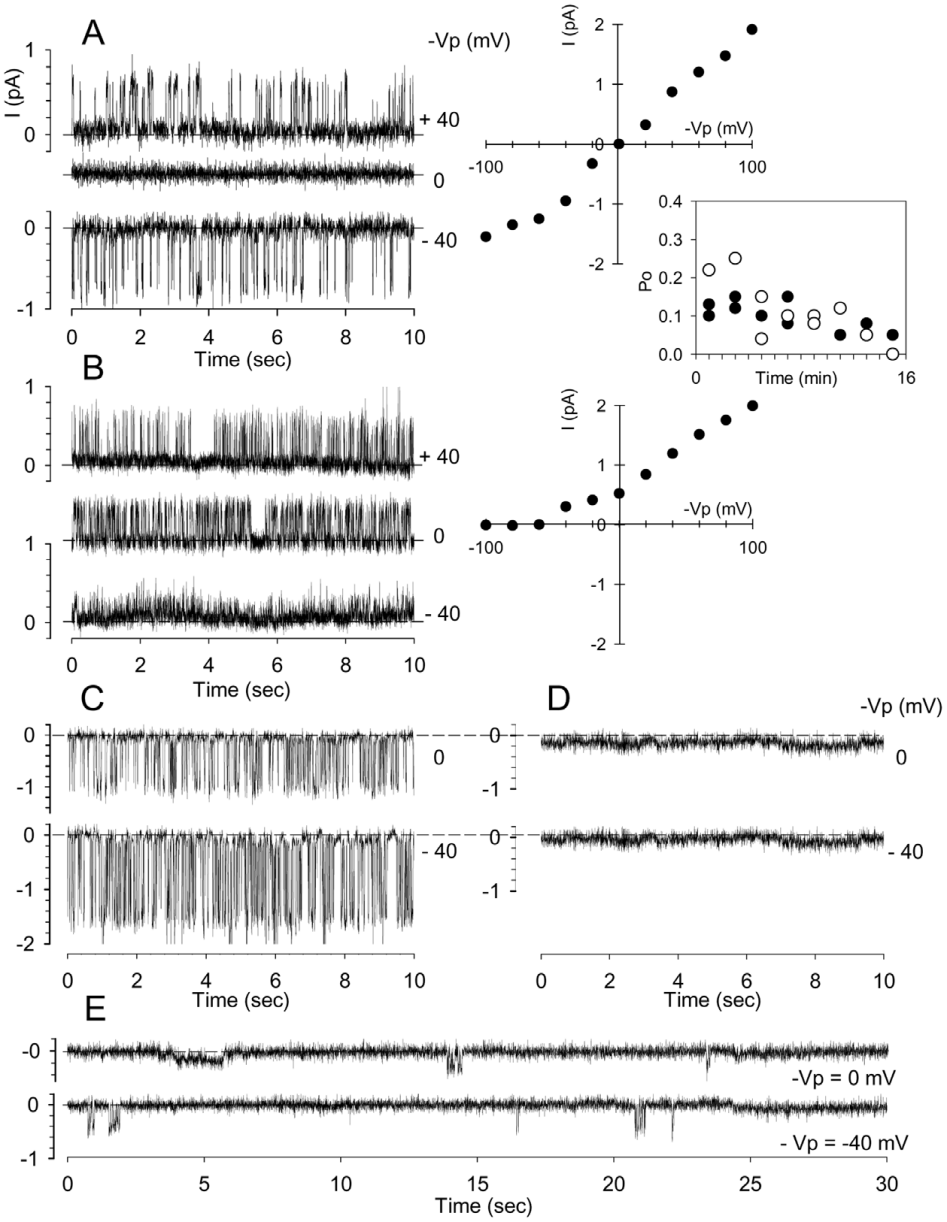
Identification of Gardos channels. (**A** and **B**) Patch-clamp single-channel recordings (representative of 25 and 19 experiments, respectively) and corresponding I/V relationships of red blood cell membrane electrical activity obtained (**A**) with solution B (150 mM KCl, pCa3) in the pipette and solution B in bath, and (**B**) with solution B in bath and solution A (150 mM NaCl, pCa3) in the pipette. The inset displays the corresponding evolution of the open probabilities (Po)(Closed symbols: panel A; open symbols: panel B). Panels **C** and **D** show, at 0 mV and −40 mV pipette potentials, inhibition of channel activity by clotrimazole (**C**: control; **D**: clotrimazole added to the bathing solution at a concentration of 10 µmol/l)(representative of 8 experiments). Requirement of extracellular calcium for activation of Gardos channels is demonstrated in Panel **E** (representative of 11 experiments) by almost total absence of channel activity whatever the imposed pipette potential (0 mV and −40 mV in the presented recordings) when the bathing solution was solution A (150 mM NaCl) adjusted to pCa7 and pipettes contained solution B (150 mM KCl) adjusted to pCa7.

Figure 4A shows current records (representative of 25 experiments) obtained with 150 mM KCl (pCa = 3) in both medium and patch-pipette resulting in symmetrical channel activity with reversal potential at 0 mV (Figure 4A, I/V curve). When the pipettes were filled with solution A (150 mM NaCl, pCa = 3) whilst the bathing solution still contained high potassium, only outward currents were observed at all imposed potentials (Figure 4B and corresponding I/V curve, representative of 19 experiments). These currents followed the direction of the K^+^ gradients (whilst chloride gradients remained constant) suggesting mediation by a K^+^-selective channel. The inset between Figure 4A and 4B displays the corresponding time-course of the open state probabilities recorded with KCl in the patch pipette (closed symbols) or with NaCl in the pipette (open symbols). Despite the wide scatter of the points in this sample, it is clear that there is a sharp late fall in Po.

Addition of the Gardos channel inhibitor clotrimazole to the bathing solution, at a concentration of 10 µmol/l (Figure 4C,4D, representative of 8 experiments), fully inhibited channel activity (Figure 4D) relative to controls (Figure 4C) whatever the voltage imposed. To investigate the Ca^2+^-dependence of the K^+^ currents it was important to reduce the Ca^2+^ concentration in the medium. With pCa = 7 in the extracellular media (bath and pipette), the success rate of gigaOhm seal formation was reduced by half. In these conditions (pCa = 7), channel activity was fully inhibited (Figure 4E, representative of 11 experiments). Absence of channel activity was also documented for the configuration pCa = 7 in bath – pCa = 3 in pipette (n = 6), whereas channel activity was retained in the configuration pCa = 3 in bath – pCa = 7 in pipette (n = 6), (data not shown). K^+^ selectivity, Ca^2+^-dependence and inhibition by clotrimazole unequivocally identify the observed currents as mediated by Gardos channels.

It is well established that Gardos channel activation requires elevated [Ca^2+^]_i_. The present experiments showed that in order to activate Gardos channels under cell-attached patch-clamp conditions it was necessary to apply a brief transient negative pressure through the patch pipette and to maintain a high Ca^2+^ concentrations in the medium, but not necessarily within the pipette. It follows then that doming of the membrane activates or generates a state of increased membrane permeability to Ca^2+^ immediately following underpressure-aided seal formation, and that the permeability increase affects membrane areas outside the patch.

**Figure 5:**
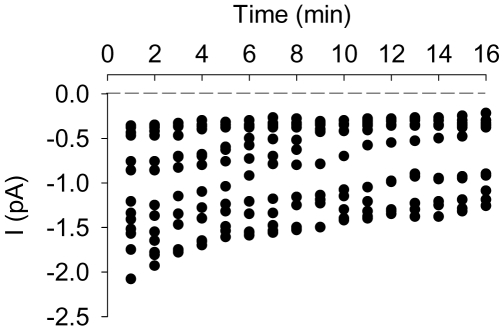
Effect of plasma membrane calcium pump (PMCA) inhibition on current amplitude as a function of time. Added to the bathing solution 2–3 min before seal formation, vanadate (1 mM) did not modify the initial diversity of current amplitudes, but the declining pattern persisted albeit at a significantly reduced rate. Following seal formation, none of the 25 patches of this series of experiments ceased electrical activity during the first 16 minutes.

A main question arising from these results concerns the transient nature of the deformation-induced response. Instant Gardos channel activation after underpressure-aided seal formation suggests a rapid initial raise in [Ca^2+^]_i_ resulting from a sudden increase in Ca^2+^ permeability (PCa). The transient nature of the response may reflect a declining time-course of PCa, a delayed response of the plasma membrane Ca^2+^ pump (PMCA) slowly restoring [Ca^2+^]_i_ to sub-activation levels of the K^+^ channels, or both. The pattern of change in open state probability documented here offers some clues. Figures 1–3 show that the decline in channel activity occurs with Po conservation, a response expected from decline in current driving gradients, to be analysed in detail in the Discussion. In all instances the fall in Po was a relatively brief terminal event. Because Po fall reflects Ca^2+^ desaturation of the channels, the sustained Po levels prior to terminal desaturation indicate that the pump-leak flux-balance within the cells must have been with [Ca^2+^]_i_ levels sufficiently high to elicit maximal, Ca^2+^-saturated channel activity. If the transient response is due to delayed pump activation then pump inhibition should lead to a sustained response. In the experiment of Figure 5, channel activity was recorded in the presence of 1 mM of vanadate which inhibits the PMCA by about 97% [26]. Added to the bathing solution 2–3 min before seal formation, vanadate did not modify the initial diversity of current amplitudes, but the declining pattern persisted albeit at a significantly reduced rate (Figure 5). None of the 25 patches in this experimental series attained full cessation of electrical activity during the 16 min following seal formation. But the declining pattern persisted suggesting that PCa spontaneously inactivates with time. PMCA activity speeds up the fall in [Ca^2+^]_i_ and consequently reduces the duration of the Gardos-mediated currents, but pump inhibition does not suppress the declining pattern. It follows that the rate of decline in the transient currents measured in the absence of pump inhibition overestimates the true inactivation rate of the deformation-induced PCa.

### Evidence for Anionic Channel Activation

**Figure 6:**
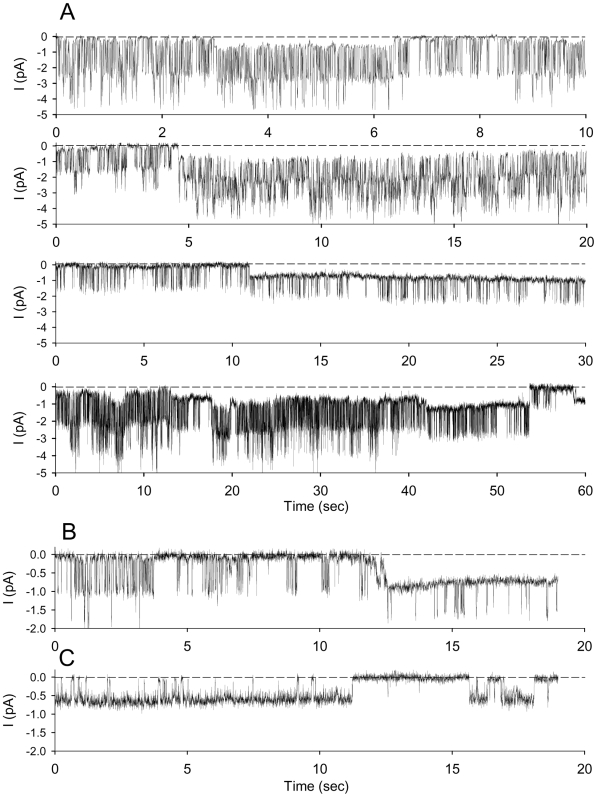
Evidence for anionic channel activation (a). Patch-clamp single-channel recordings of red blood cell membrane electrical activity obtained during the first 10 min following seal formation in absence of pipette potential (Vp = 0 mV). (**A**) shows four different recordings, obtained with solution B (150 mM KCl, pCa3) in the pipette and solution A (150 mM NaCl, pCa3) in bath. The sudden shifts of the baseline underlying the K^+^ channel current reflects the onset of an additional type of channel activity. Panel **B** presents a sample of recording where the Gardos channel and an anionic channel are present simultaneously and the recording displayed in **C** (representative of 12 experiments) shows, on the same patch, that after inhibition of Gardos channel activity by clotrimazole, added in the bathing solution at the concentration of 10 µmol/l, the remaining anionic activity was characterized by long openings followed by long closure intervals.

**Figure 7:**
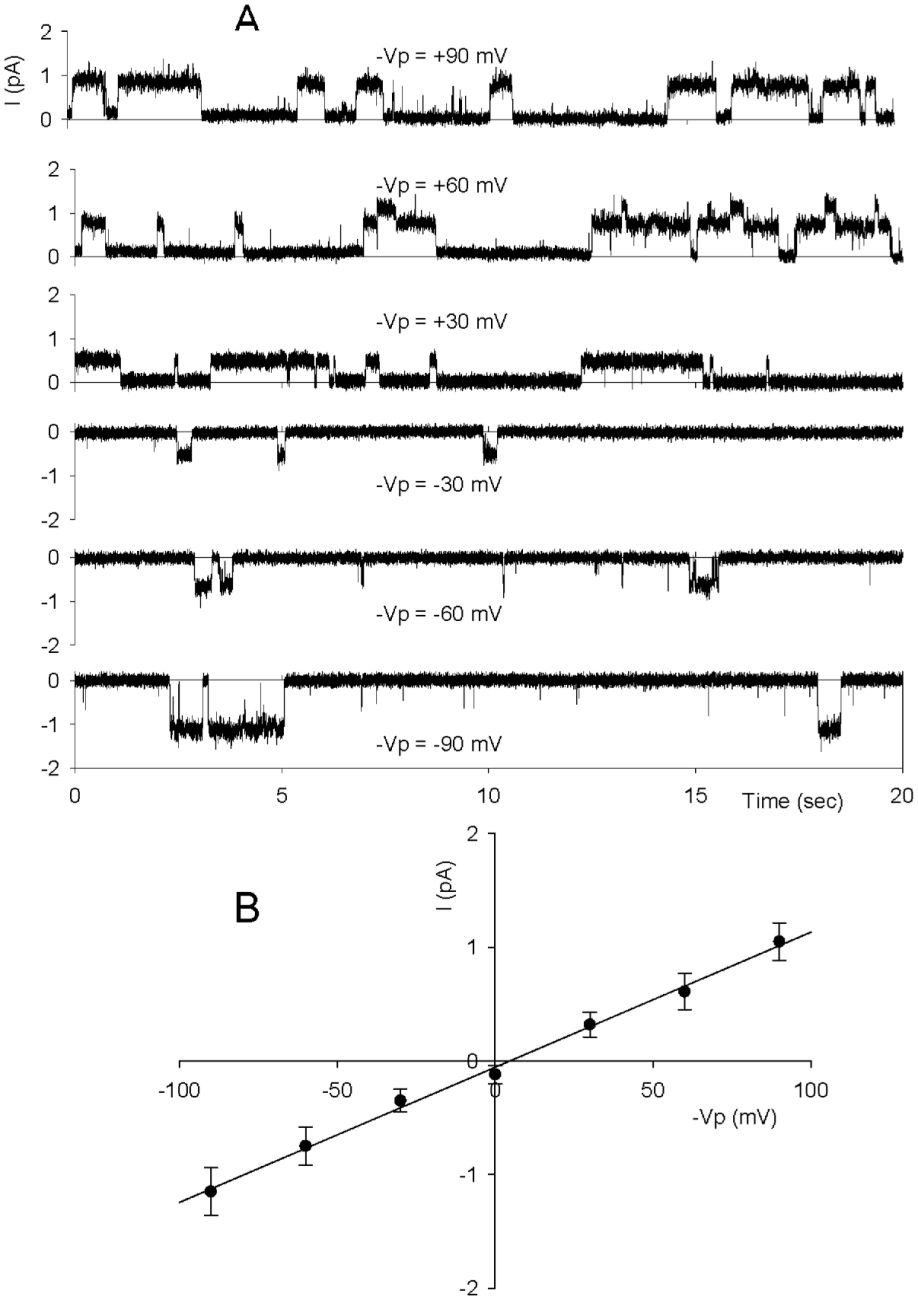
Evidence for anionic channel activation (b). An example of patch-clamp single-channel recordings obtained during the first 10 min following seal formation at at different membrane potentials (Panel **A**), with solution A (150 mM NaCl, pCa3) in the pipette and bathing solutions, and corresponding I/V relationship (Panel **B**). Points are means ± SEM obtained from 6 experiments.

As illustrated in Figure 1B, with KCl in the patch-pipette, Gardos channel activity was always observed immediately after seal formation. On the other hand, with NaCl in the patch pipette (Figure 1A), when K^+^ currents are not detected across the patch, inward currents were frequently observed at the spontaneous membrane potential, characterized by long openings followed by long closure intervals. The records in Figure 6A, obtained with KCl in the patch pipette, show four characteristic examples in which the baseline K^+^ channel current was suddenly shifted due to additional current resulting from the onset of another type of channel activity. These cell-attached recordings were obtained at the spontaneous membrane potential in the absence of any imposed pipette potential (-Vp = 0 mV). The magnitude of this current varied slightly from one patch to another and declined with time in a similar manner to the K^+^ channel activity, but the range of these variations remained much smaller (−0.5 mV to −1 pA). The sudden baseline shifts did not occur in cells treated with NPPB prior to patch-clamping (data not shown). Figures 6B and 6C (representative of 12 experiments) are sequential records from the same patch, before (Figure 6B) and after (Figure 6C) addition of clotrimazole (10 µM) to the bathing solution. The remaining activity after Gardos channel inhibition by clotrimazole displayed alternating long intervals of open-closed states. In another series of experiments, illustrated in Figure 7, use of NaCl (Solution A) in pipette and bathing solutions allowed stable recordings of channel activity 10 min after seal formation, at different imposed potentials. The channel activity recorded in these conditions was inhibited by NPPB and DIDS (not shown) and can be clearly identified as an anionic current. The I/V curve (Figure 7B) shows a linear conductance over the ±100 mV range, with an 11.5 pS slope (n = 6). Collectively, these results reveal an anionic current component secondary to, and induced by Gardos channel activation.

## Discussion

The aim of the work reported here was to elucidate the mechanism of a transient current response encountered during patch clamping of human red blood cells using the cell-attached configuration. The results provided the first direct electrophysiological evidence of Gardos channel activation in intact red blood cells by the sole effect of a local membrane deformation, and of the participation of secondarily activated anionic currents in the deformation-triggered response. We analyse first the mechanism of the transient response and consider next the implications of our findings to the understanding of red blood cell homeostasis.

### The Role of Ca^2+^ and the Transient Nature of the Current Response

Gardos channel activation required the presence of calcium in the medium but not in the patch pipette suggesting that local deformation somehow activated Ca^2+^ permeability pathways, PCa, distant to the domed area within the patch pipette, leading to increased [Ca^2+^]_i_. The powerful, ATP-fueled plasma membrane Ca^2+^ pump maintains a physiological pump-leak balance with an intracellular Ca^2+^ concentration in the 20–50 nM range [27], well below Gardos channel activation. The instant Gardos channel-mediated response observed upon membrane deformation under the patch pipette (Figures 1 and 2A), suggests that calcium entering the cell via PCa, driven by the steep inward Ca^2+^ gradient, induces a new pump-leak balance with [Ca^2+^]_i_ high enough to elicit maximal activation of the Gardos channels. The results showed that at the temperature of our experiments (35°C±2°C) the open state probability of the channels during the first ∼ten minutes was in the range 0.3 to 0.4 (Figure 2B). This range is far above the 0.01–0.1 values reported by Grygorczyk [28] at 35°C in excised inside-out patches at saturating Ca^2+^ concentrations [29], [30], suggesting that the excised inside-out configuration does not represent the physiological response of Gardos channels in the intact cell, perhaps because of the lack of essential cytoplasmic regulatory factors such as calpromotin [31].

Two distinct phases characterized the transient response. Firstly, a long initial period in which current amplitude was reduced at constant high Po (analysed separately in the section below). Secondly, a relatively brief terminal period with a sharp decline in Po, reflecting Ca^2+^ desaturation of the Gardos channels. When the Ca^2+^ pump was inhibited by vanadate (Figure 5), the duration of both phases increased, but the response remained transient, indicating that residual pump activity was still effective in balancing a declining PCa to [Ca^2+^]_i_ levels below Gardos channel activation. Thus, the understanding derived from these results clearly attributes the transient nature of the response to the spontaneous decline in the PCa elicited by the initial deformation.

Another feature of the transient response was the variability in its duration, as apparent in Figures 2C and 5. Early work documented a large variability in maximal Ca^2+^ extrusion capacity by the pump (*V*_max_) among cells [11], [14]. This variability was recently correlated with cell age, older cells having much weaker pumps than younger cells [32]. Therefore, for a similar spontaneous declining time-course in deformation-induced PCa, a young cell with a high pump *V*_max_ will be able to restore [Ca^2+^]_i_ down to sub-activation levels for Gardos channels faster than an older cell.

**Figure 8:**
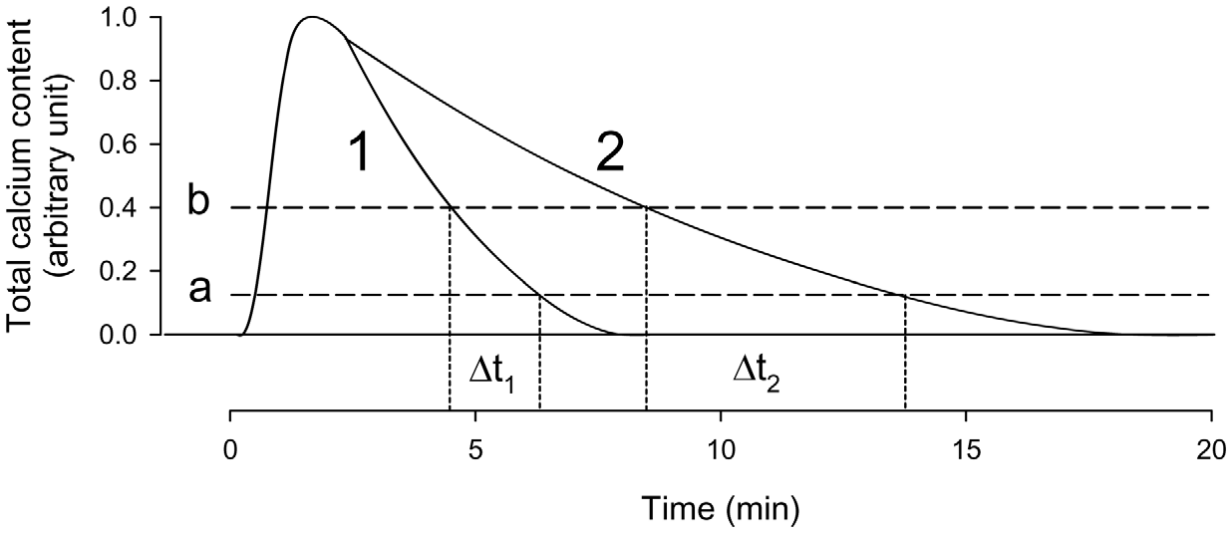
Diagrammatic representation of hypothesized time course of changes in total cell calcium. Upon membrane deformation, calcium enters the cell via PCa driven by a steep inward Ca^2+^ gradient. A new pump-leak balance is generated with elevated [Ca^2+^]_i_ initially set at an arbitrary maximal level of total calcium content (1.0 arbitrary unit). As PCa declines with time, the changing pump-leak balance is with a time-declining pattern in total cell calcium. PMCA *V*_max_ declines with RBC age, so that the time course of Ca^2+^ extrusion will be faster in young cells (curve 1) than in old or pump-inhibited cells (curve 2). Dashed line (***a***) represents the threshold of Gardos channel activation. Dashed line (***b***) represents the calcium level above which Gardos channel remains Ca^2+^-saturated with maximal activity (maximal Po). During the process of Ca^2+^ extrusion with declining PCa, the time spent between levels (***b***) and (***a***) varies with cell age. During the intervals Δt_1_ and Δt_2_ the open state probability of the Gardos channels declines progressively to extinction following Ca^2+^ desaturation.

This interpretation is illustrated in Figure 8, in which a normalized increase in total cell calcium content is plotted as a function of time for the duration of the cell-attached protocol. The solid lines in curves 1 and 2 describe the hypothesized biphasic pattern of change in total cell calcium. The sharp initial deformation-induced rise after PCa activation is followed by a progressive reduction resulting from the changing pump-leak balance at each instant of time during the spontaneous decline in PCa. Line ***a*** represents a [Ca^2+^]_i_ threshold level for Gardos channel activation below which the channels remain silent. Line ***b*** represents the [Ca^2+^]_i_ saturation level above which Gardos channel activity is maximal. Thus, while the Ca^2+^ content is above ***b*** Po remains constant at it maximal level. Between ***b*** and ***a***, Po becomes progressively reduced following Ca^2+^ desaturation. With this representation, it is possible to explain the observed variability in the duration of the transients by the variable pump-mediated calcium extrusion capacity among the cells, young cells represented by curve 1, older cells or pump-inhibited cells (Figure 5) represented by curve 2. The corresponding duration of the desaturation periods during which Po becomes progressively reduced is indicated by Δt1 and Δt2. Finally, it should be stressed that although pump-variability can explain the observed results, these do not rule out a contribution from differences in PCa decline-rates among cells,

### Analysis of Red Blood Cell Homeostasis and Ionic Currents during Deformation-Induced PCa

**Figure 9:**
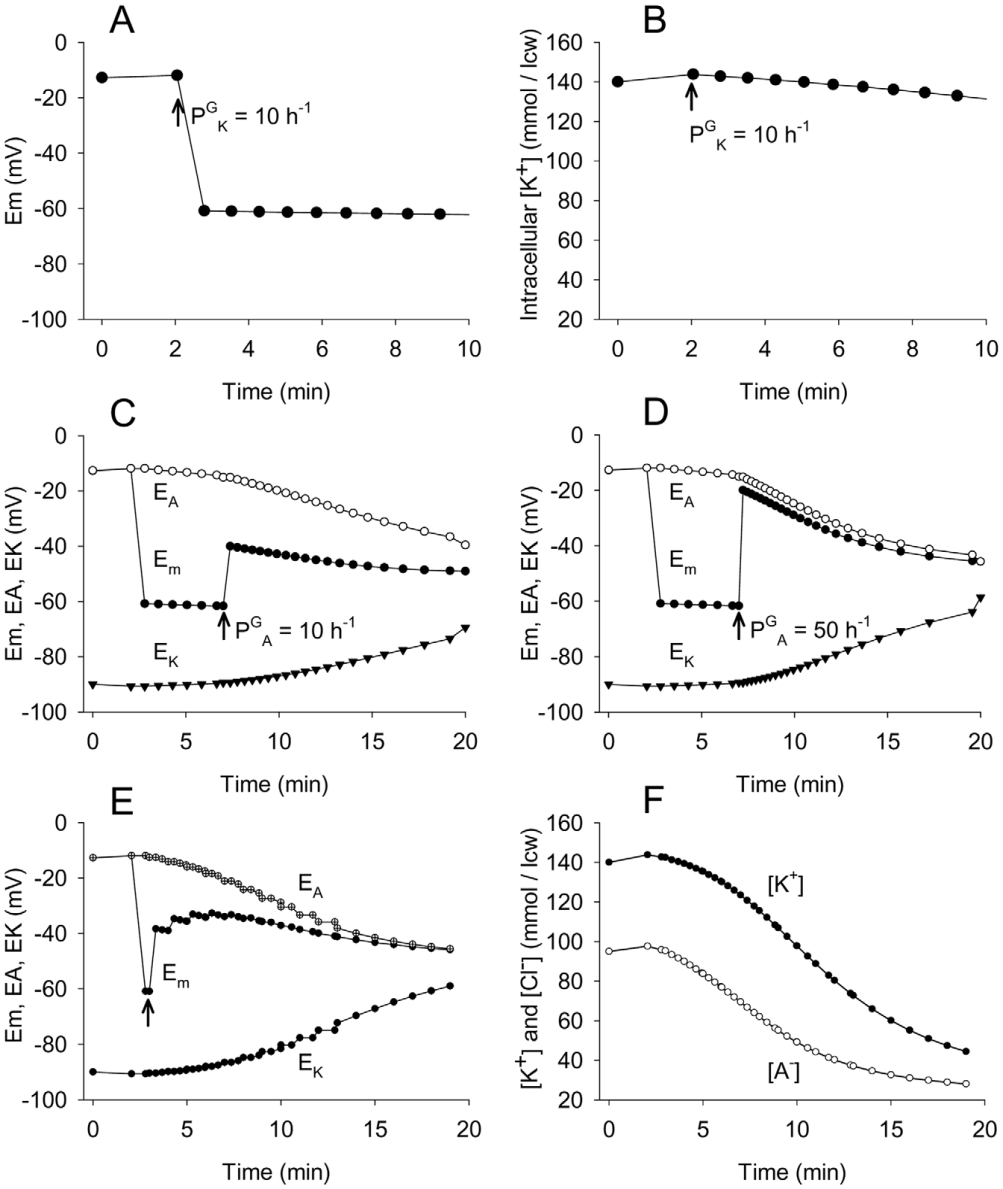
Analysis of the homeostasis of a red blood cell under cell-attached patch clamp with the use of the Lew-Bookchin model. The predicted effects of sudden and maximal Gardos channel activation on the membrane potential Em and intracellular K^+^ concentration ([K^+^]) are presented in panels **A** and **B**. Electrodiffusional permeability for K^+^ ions (P^G^_K_) was increased to a value of 10 h^−1^ at time t = 2 min. The parameter values chosen for this simulation were 5 mM KCl and 150 mM NaCl in external bathing solution. The initial P^G^_K_ value was 0.001651 h^−1^; for other steady state default parameters refer to Lew and Bookchin [34]. Panels C and D present the simulated effects on Em, EK (equilibrium potential for K^+^ ions) and EA (equilibrium potential for anions) of sudden and maximal Gardos channel activation (as above) followed by sudden activation of anionic electrodiffusional permeability (P^G^_A_). At time t = 7 min, P^G^_A_ was changed from 1.2 h^−1^ to 10 h^−1^ (**C**) or 50 h^−1^ (**D**) corresponding to moderate and large activations. To simulate the effects of a gradual increase in anionic electrodiffusional permeability (panel **E**), P^G^_A_ was changed from 1.2 h^−1^ to 5 h^−1^ at t = 3 and incremented by 5 h^−1^ each min to reach P^G^_A_ = 35 h^−1^ at t = 18 min). Panel **F** displays the corresponding evolution of intracellular K^+^ and A^−^ concentrations.

The observed reduction in current amplitude without variation in open-state probability cannot be attributed to Gardos channel Ca^2+^ deactivation [33]. In high-K^+^ media, the results showed no reduction in current amplitude with time, only the brief terminal phase of Po decline (Figure 4). Current amplitude fall at constant Po must therefore reflect a progressive reduction in electrochemical driving force for K^+^ movement through the Gardos channels. To analyse this whole-cell response we used the integrated Lew-Bookchin red cell model [34]. The time change in the relevant homeostatic variables was followed with different simulated experimental protocols exploring which changes in cell permeability and electrochemical driving forces could explain the measured current amplitude decline. The results are shown in Figure 9. The initial two-minute pre-patch period in each simulation records the minor changes induced by transferring the model cell from its default-defined reference state to the new extracellular medium in each simulated condition. The variables reported are: membrane potential (Em), intracellular K^+^ and Cl^−^ concentrations ([K^+^], [Cl^−^]), and the equilibrium potentials for K^+^ and Cl^−^ (E_K_ and E_A_; we use A^−^ to signify any monovalent anion with permeability similar to that of Cl^−^, HCO_3_^−^ for instance). The effect of membrane deformation was simulated by a sudden and sustained increase in K^+^ permeability (P^G^_K_) to 10 h^−1^, a value representing the mean increase in K^+^ permeability measured in red blood cell suspensions with maximally activated Gardos channels [29], [35].

Increasing P^G^_K_ caused immediate and sustained hyperpolarization, from −12 mV to −60 mV (Figure 9A). In these conditions, Em approaches E_K_ (Figure 9C) and [K^+^] declines minimally with no significant change in driving force for net K^+^ efflux. Therefore, Gardos channel activation per se cannot account for the observed decline in the amplitude of the K^+^ currents. This simulation accurately describes the slow rate of net K^+^ loss following Gardos channel activation in experiments with RBC suspensions [29], [36], reflecting the rate-limiting effect of the relatively low electrodiffusional anion permeability (P^G^_A_). However, our results show that upon deformation-induced Gardos channel activation, anion-selective channels also become activated (Figures 6 and 7). Thus, the electrodiffusional anion permeability pathway of RBCs seems to respond differently when the RBCs are free in suspension and P^G^_A_ remains unchanged by Gardos-mediated hyperpolarization, and when individual cells are locally deformed under the patch, as if the local deformation that activated PCa could also alter the normal response of endogenous anionic channels.

The simulations in Figures 9C-9F explore the effects of increased P^G^_A_ following P^G^_K_ activation. When P^G^_A_ activation was simulated 5 min after the onset of P^G^_K_, the model predicted large instant reductions in P^G^_K_-induced hyperpolarization (Figures 9C and 9D) with Em approaching E_A_, closer the higher the induced P^G^_A_. If P^G^_A_ is increased gradually after P^G^_K_, the initial hyperpolarization declines gradually towards E_A_ (Figure 9E) and the intracellular concentrations of K^+^ and Cl^−^ decline markedly with time. Thus, the effects of a progressive increase in P^G^_A_, as simulated in Figures 9E and 9F, offer a plausible explanation for the declining amplitude of the Gardos-mediated currents, in line with the observed changes in K^+^ and Cl^−^ currents.

### Current Flows Under the Cell-Attached Patch-Clamp Configuration in Red Blood Cells

A minimum of one copy of the Gardos channel was present under the pipette tip, and it was not unusual to count as many as four copies simultaneously open. Assuming uniform surface density of Gardos channels, and between 150 and 300 copies per cell [37], [38], [39], we can estimate that up to about three percent of membrane area became accessible for current recordings within the domed patch upon seal formation.

**Figure 10:**
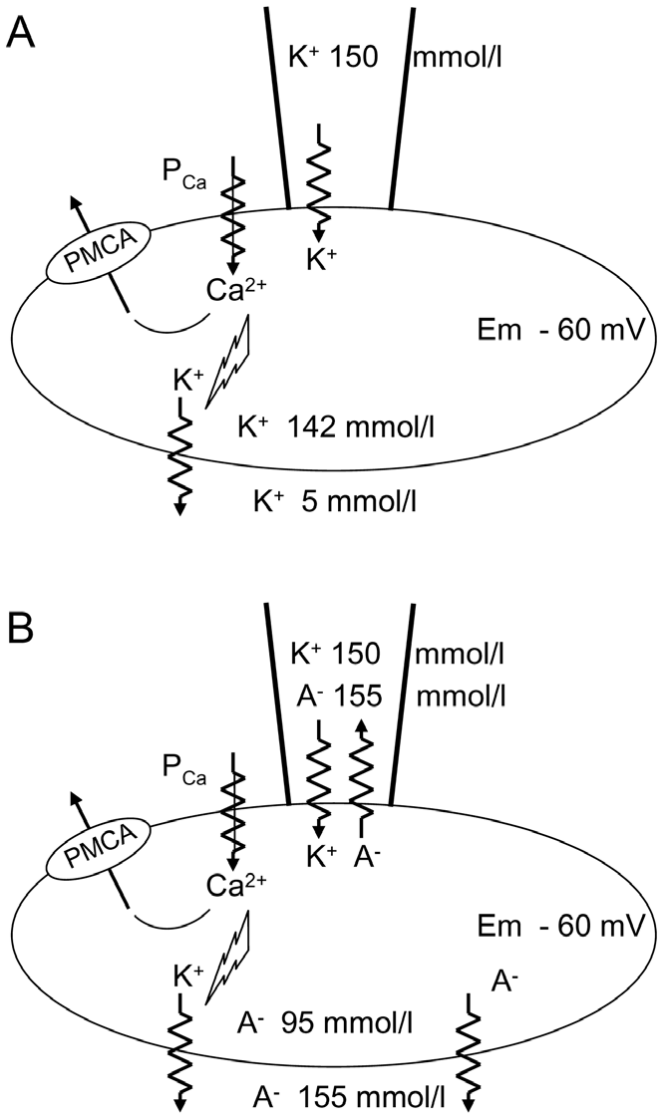
Diagrammatic representation of the currents and transporters involved in the deformation-induced transient response. Panel (**A**). Upon membrane deformation, calcium enters the cell driven by a steep inward gradient; the consequent elevated [Ca^2+^]_i_ activates Ca^2+^-sensitive K^+^ channels. The resulting hyperpolarization increases the driving force for inward movement of K^+^ ions from the high-K^+^ pipette solution to the cell interior. Because the three compartments, extracellular medium, cell interior and patch pipette are in series, with low-K^+^ in the extracellular compartment the K^+^ fluxes are oriented from the pipette to the cell and from the cell to the extracellular compartment. Elevated [Ca^2+^]_i_ at the inner membrane surface stimulates uphill Ca^2+^ extrusion through the PMCA. Panel (**B**). Hyperpolarization also sets the driving force for net anion (A^−^) loss through electrodiffusional pathways. The small chemical gradient which exists between the bath/pipette (155 mM) and the cell interior (95 mM) is largely offset by Gardos channel-mediated hyperpolarization, resulting in outward movement of anions both at pipette and whole cell levels.

The cell-attached patch configuration generates three compartments in series: patch pipette, cell interior, and extracellular medium. The membrane potential under the cell-attached pipette always follows very closely the whole-cell membrane potential [40]. With high-K^+^ within the patch pipette and low-K^+^ in the extracellular medium, Gardos channel mediated hyperpolarization increases the driving force for inward movement of K^+^ ions from the pipette solution to the cell interior. The K^+^ fluxes are oriented from the pipette to the cell and from the cell to the extracellular compartment. These K^+^ flows are illustrated in Fig. 10A. Extracellular high-K^+^ prevented hyperpolarization and net K^+^ current flow between extracellular medium and cell interior.

It might seem surprising that the inward current recorded through the membrane patch, corresponding to anionic electrical activity (Figures 6 and 7) reflected an exit of anions from the cell to the pipette, whilst at the same time the flux of anions followed the flux of K^+^ ions through the rest of the red cell membrane (Figure 10B). In the absence of imposed potentials, the slight chemical gradient of Cl^−^ existing between bath and pipette with 155 mM, and cell interior with 95 mM, was largely overcome by the electrical gradient resulting from Gardos channel activation driving an outward movement of anions from the cell. The asymmetric behaviour of the ion fluxes from pipette to cell, and cell to bath is a consequence of the asymmetric Nernst potentials for potassium. In both cases the ions are simply moving down their gradient.

### Conclusions

The experiments reported here show that a local membrane deformation under a cell-attached patch elicits generalized but transient membrane permeability changes in human red blood cells. The increased Ca^2+^ permeability away from the deformed patch elevates [Ca^2+^]_i_ and activates K^+^ currents through Gardos channels and secondary anion currents through NPPB and DIDS-inhibitable channels. These findings expose unexpected RBC responses, with participation of anion permeability changes never seen before in experiments with red blood cell suspensions, of potential relevance to RBC behaviour in the circulation in vivo. Thus, a local membrane deformation able to trigger a transient increase in Ca^2+^ permeability with secondary activation of K^+^ and anionic channels has the potential to induce significant dehydration even during a brief deformation event in the microcirculation, an important new contributing factor for consideration in the pathophysiology of sickle cell dehydration and in the normal mechanism of densification of aging red blood cells.

## Materials and Methods

### Chemicals

Clotrimazole, 4,4′-diisothiocyanostilbene-2,2′-disulphonic acid (DIDS), N-methyl-D-glucamine chloride (NMDG) 5-Nitro-2-(3-phenylpropylamino)-benzoate (NPPB), ethylene glycol-bis(2-aminoethylether)-N,N,N, N,-tetraacetic acid (EGTA), were purchased from Sigma (Saint Quentin Fallavier, France).

### Solutions

Basic pipette and bathing solutions for cell-attached configuration (solution A) contained (in mM): 150 NaCl, 5 KCl, 1 MgCl_2_, 10 Hepes, 10 glucose, pH 7.4. For identification of K^+^ channels, in cell-attached or excised inside-out configuration, pipette and/or bathing solutions contained (in mM) 150 KCl, 5 NaCl, 1 MgCl_2_ 10 Hepes, pH 7.4 (solution B).

EGTA was used as Ca^2+^ buffer when the pCa (-Log[Ca^2+^]) was adjusted to pCa = 7. All solutions were filtered through 0.2 µm Millipore cellulose disks and equilibrated in air (oxygen partial pressure, pO_2_ = 155 mmHg; carbon dioxide partial pressure, pCO_2_ = 0.3 mmHg). Osmolality was 310±5 mosmolal.

### Preparation of Cells

Venous blood was drawn into heparinized vacutainers from healthy volunteers upon written informed consent. RBCs were washed thrice by centrifugation and resuspension in large volumes of culture RPMI medium (Gibco BRL). The buffy coat containing platelets and white cells was removed by suction after each wash. After the last wash, the cells were suspended at 50% hematocrit in RPMI and kept at 4°C. They were suspended at 10% hematocrit in solution A and incubated at 37°C for about 1 hour prior to experiments. Experiments were done at the temperature of 35±2°C.

### Current Recordings

Patch pipettes (tip resistance 10–20 MOhm) were prepared from borosilicate glass capillaries (GC 150F Clark Electromedical), pulled and polished on a programmable puller (DMZ, Werner Zeitz, Augsburg, Germany). Doming patch-clamp seals (4 to 20 GOhm) were obtained by a suction pulse of 10 mmHg applied for less than 5 s, using a syringe connected to the patch pipette. Single channel currents were recorded using a RK400 patch-clamp amplifier (Biologic, Claix, France), filtered at 0.3 or 1 kHz, digitized (48 kHz) and stored. For analysis, played back data was processed by the WinEDR V2.7.6 computer program (Dempster, Strathclyde Electrophysiology Software).

The sign of the clamped voltage (Vp) refers to the pipette solution with respect to the bath. Outward currents (positive charges flowing across the patch membrane into the pipette) are shown as upward deflections in the current traces. In the cell-attached configuration the imposed membrane potential (Vm) is referred to as -Vp. Current-voltage (I/V) curves were constructed by plotting the mean current amplitude for each clamped potential. Open probability (Po) was determined as the fraction of digitized points above a threshold set midway between the closed and open peaks of current-amplitude histograms. Each Po was determined from 60 seconds stable recordings. In these conditions, Po was defined as the ratio of the total time spent in the open state to the total time of the record. Analyses were confined to patches containing one channel event histogram. Data are given as mean values ± SEM. The Lew-Bookchin mathematical-computational model [34] of the homeostasis of human RBC is available as a free-standing executable file from http://www.physiol.cam.ac.uk/staff/lew/index.htm.
